# Carboxylated Cellulose Nanocrystals Decorated with Varying Molecular Weights of Poly(diallyldimethylammonium chloride) as Sustainable Antibacterial Agents

**DOI:** 10.3390/polym15040865

**Published:** 2023-02-09

**Authors:** Eliskander Rabia, Beza Tuga, José de Ondarza, Saleen M. Ramos, Edmond Lam, Sabahudin Hrapovic, Yali Liu, Rajesh Sunasee

**Affiliations:** 1 Department of Chemistry and Biochemistry, State University of New York at Plattsburgh, Plattsburgh, NY 12901, USA; 2 Department of Biological Sciences, State University of New York at Plattsburgh, Plattsburgh, NY 12901, USA; 3 Aquatic and Crop Resource Development Research Centre, National Research Council Canada, Montreal, QC H4P 2R2, Canada; 4 Department of Chemistry, McGill University, 801 Sherbrooke St. West, Montreal, QC H3A 0B8, Canada

**Keywords:** cellulose nanocrystals, cationic polymer, synthesis, characterization, antibacterial

## Abstract

Cationic nanomaterials are promising candidates for the development of effective antibacterial agents by taking advantage of the nanoscale effects as well as other exceptional physicochemical properties of nanomaterials. In this study, carboxylated cellulose nanocrystals (cCNCs) derived from softwood pulp were coated with cationic poly(diallyldimethylammonium chloride) of varying molecular weights. The resulting cationic carboxylated cellulose nanocrystals coated with poly(diallyldimethylammonium chloride) (cCNCs–PDDA) nanomaterials were characterized for their structural and morphological properties using Fourier transform infrared spectroscopy, dynamic light scattering, zeta potential, elemental analysis, transmission electron microscopy, and thermogravimetric analysis. Cationic cCNCs–PDDA were investigated for their antibacterial properties against Gram-positive *Staphylococcus aureus* and Gram-negative *Escherichia coli 23934* and *Pseudomonas aeruginosa* using a bacterial lawn growth inhibition assay. cCNC–PDDA materials displayed marked antibacterial activity, particularly against Gram-positive *Staphylococcus aureus*. Overall, our results indicated that cCNCs–PDDA could be a potential candidate for antibacterial applications such as antibacterial surfaces or coatings.

## 1. Introduction

Bacterial infections remain a major cause of morbidity and mortality and continue to have a large impact on public health [1,2]. Despite the existence of potent antibiotics to fight bacterial infections, resistant bacterial strains are still on the rise. As such, there is still a strong demand for developing novel and effective antibacterial agents, particularly nanomaterial-based antibacterial agents. Recent studies on the use of nanomaterials or surface-modified nanomaterials as antibacterial agents have shown great promise in combating bacterial infections that are usually difficult to treat and/or have the ability to evade existing mechanisms linked with acquired drug resistance [3,4,5]. Nanomaterials such as metal-based nanoparticles [6,7,8,9], polymeric nanostructures [10,11,12], and carbon-based nanomaterials [13] have displayed antibacterial activity against several pathogenic bacteria with tremendous potential as bactericidal materials. In addition, several key factors for controlling the antibacterial activity of nanomaterials have been identified for a precise and intelligent design of nanomaterial-based antibacterial agents to address bacterial infections. These include their dimensions, element composition, shape selection, modification density surface charge, and topography [14]. All these factors are crucial for regulating the first interaction between nanomaterials and bacteria. For instance, the shape and size of nanomaterials can affect their entry into bacterial cells. Furthermore, the charge effect can influence the binding of the nanomaterial to the bacterial surface which is the initial step of the entry of the nanomaterial into the bacterial cells. Since bacterial surfaces are endowed with a negative charge, the design of nanomaterials possessing cationic groups makes them ideal candidates to help with the adhesion of nanomaterials to bacteria [14]. Among carbon-based nanomaterials such as carbon nanotubes, fullerene, nanodiamonds, oxide nanosheets, etc. [13], nanocelluloses have emerged as a unique class of nanomaterials for the smart design and fabrication of bactericidal materials. Nanocelluloses are nanoscale forms of cellulose and hence combine both the features of cellulose and nanomaterials. Nanocelluloses include bacterial nanocelluloses (BNC), cellulose nanofibrils (CNFs), and cellulose nanocrystals (CNCs) [15,16,17]. They all vary in their production methods, with BNC being produced biotechnologically by bacteria while CNFs are isolated by the mechanical delamination of plant-based cellulose. CNCs, on the other hand, are commonly produced by chemical methods such as acid hydrolysis or oxidation.

CNCs are one of the most abundant natural biopolymers on earth that are derived from the acid hydrolysis of cellulose. Concentrated sulfuric acid is a commonly used acid for the hydrolysis of native cellulose (typically wood pulp and other various cellulosic sources) to obtain stable colloidal suspensions of negatively charged sulfated CNCs. CNCs are promising nanoplatforms for the development of antibacterial nanomaterials due to their sustainability, biocompatibility, biodegradability, and unique physicochemical properties [15,16,17]. Furthermore, CNCs possess a rod-like morphology with a high surface area-to-volume ratio (compared to spherical particles) and a surface richly endowed with hydroxyl groups for chemical modifications. While CNCs do not intrinsically display antimicrobial properties, surface functionalization with antimicrobial agents has been strategized as a fabrication method for antimicrobial CNCs-based hybrid materials. Covalent or non-covalent surface chemical modifications of CNCs impart new properties to the surface of the nanocrystals and the CNC size dimensions and surface charges can easily be tuned accordingly [18,19]. Previous works on CNC-based antibacterial agents included the use of CNC–inorganic hybrids (ZnO, TiO_2_, and Ag_2_O) which displayed effective antibacterial properties against *Escherichia coli* and *Staphylococcus aureus* [20,21]. In a recent study, CNCs in powdered form were used as a dispersing agent for the preparation of antimicrobial spray-dried nanoparticle-in-microsphere formulations with aluminum oxide nanoparticles, zinc oxide, and zinc oxide nanoparticles [22]. The formulations displayed potent antimicrobial and antibiofilm activity against Gram-negative and Gram-positive bacteria. The antimicrobial activity of nanoparticle-in-microsphere was found to be higher than that of each raw component. The highest antimicrobial and antifungal activity was obtained with a formulation consisting of CNCs (weight % ≥ 0.75%) and an equal weight % of aluminum oxide and zinc oxide NPs (~0.25% per type of nanoparticle). For less toxic and more sustainable CNCs-based antibacterial agents, some organic antimicrobial agents, such as chitosan [23,24], curcumin [25], carvacrol [26], rosin [27] and allicin/lysozyme [28] were incorporated on CNCs and tested against several bacteria [29]. Since cationic functionality is known to be responsible for bacterial killing due to the strong interaction of the cationic groups with the bacterial cells [30], CNCs were also conjugated with cationic organic molecules that displayed antibacterial properties. For example, cationic porphyrin was covalently linked to the surface of CNCs using the Cu(I)-catalyzed Huisgen–Meldal–Sharpless 1,3-dipolar cycloaddition (click chemistry). CNCs were first chemically modified in two steps to incorporate surface azide groups which subsequently reacted with an alkyne-containing porphyrin under click reaction conditions. The cationic CNC–porphyrin materials were effective in the photodynamic inactivation of *Mycobacterium smegmatis* and *Staphylococcus aureus*, but showed only slight efficacy against *Escherichia coli*. The photobactericidal porphyrin-CNCs have potential applications in the healthcare and food preparation industries [31]. Bespalova et al. reported a two-step synthesis to generate CNCs modified with quaternary ammonium organic molecules of varying alkyl chain length [32]. Using the disc-diffusion method, they found that modified CNCs with alkyl chains longer than ten carbons were effective antimicrobial agents against *Staphylococcus aureus* and *Escherichia coli*. In another study, Tang et al. fabricated polyrhodanine-coated CNCs using a simple green polymerization approach. Rod-like polyrhodanine-coated CNCs displayed promising antimicrobial properties toward both *Escherichia coli* and *Bacillus subtilis* that were comparable to spherical nanocomposite particles. The authors proposed that this could be due to the lower percolation threshold for rod-like nanoparticles resulting from the higher aspect ratio [33]. 

Most examples to date have used sulfated CNCs as support for bioactive materials, whether they are organic or inorganic in nature. There are still limited studies on the design of cationic CNC-based antibacterial agents and hence, in this work, we explored the design, synthesis, characterization, and antibacterial properties of wood-based carboxylated CNCs (cCNCs) coated with the cationic polymer, poly(diallyldimethylammonium chloride) (PDDA). cCNCs were prepared via an oxidation method using ammonium persulfate (APS) as a mild, low-cost, and low-toxicity oxidant [34]. An advantage to the APS process is its versatility in converting different lignocellulosic biomass into cCNCs without the need for pre-treatments of the starting biomass. In this oxidation reaction, persulfate anions become free radicals and hydrogen peroxide that can hydrolyze amorphous cellulose, decolorize aromatic components of plant material, and oxidize the C6 alcohol groups on the surface of the nanocrystal to yield negatively charged carboxylate groups. It is these carboxylate groups that promote the electrostatic repulsion between neighboring cCNCs and thus avoid aggregation and form colloidally stable solutions, with comparable nanoscale geometries and crystallinity to sulfated CNCs. The negatively charged surface of the cCNCs will also allow for the non-covalent binding of cationic materials on the surface of cCNCs. PDDA possesses the quaternary ammonium moiety as a pendant cationic group in its chemical structure and with a good safety profile for antimicrobial or medical applications and food processing [35,36]. To the best of our knowledge, the antibacterial properties of PDDA-functionalized cCNC materials have not been reported. Herein, the surfaces of cCNCs were coated with three different molecular weights (MWs) of PDDA, 8500 (low MW), 240,000 (intermediate MW), or 400,000–500,000 (high MW), using a one-step non-covalent synthetic method. The antibacterial properties of the resulting cationic cCNC–PDDA materials were assessed against three bacterial strains, namely, *Staphylococcus aureus* (SA), *Escherichia coli* (EC), and *Pseudomonas aeruginosa* (PA), using a bacterial lawn growth inhibition assay. The effect of the MW (low, intermediate, and high) of the PDDA decorated onto cCNCs on the antibacterial properties was also investigated. The results indicated that these cationic cCNCs–PDDA displayed antibacterial effects against SA, EC, and PA with the SA being the most affected. Cationic cCNCs–PDDA could be potential candidates for antibacterial applications, such as antibacterial surfaces or coatings. 

## 2. Materials and Methods

### 2.1. Materials and Reagents

Reagent grade poly(diallyldimethylammonium chloride) (PDDA) at 28 wt% in water with MWs of 8500 and 240,000 were purchased from Polysciences, Inc. (Warrington, PA) while PDDA with MWs of 400,000–500,000 and sodium chloride were purchased from Sigma Aldrich and used as received. *Staphylococcus aureus* (SA), *Escherichia coli 23934* (EC), and *Pseudomonas aeruginosa* (PA) were obtained from Presque Isle Cultures (Erie PA).

### 2.2. Preparation of cCNCs and cCNCs Coated with Different Molecular Weights of PDDA

#### 2.2.1. Preparation of cCNCs

Carboxylated CNCs (cCNCs) were prepared via a one-step APS oxidation method [37,38]. Briefly, softwood pulp (3 kg) was added to 1 M ammonium persulfate solution (200 L). The mixture was then heated to 60 °C for 16 h to afford a suspension of cCNCs which underwent decantation, centrifugation (12,000 rpm, relative centrifugal force = 22,100 for 10 min), and diafiltration to a working volume of 30 L to remove reaction by-products. The isolated cCNCs were then neutralized to pH 7 with 1 M NaOH before dispersion using a homogenizer, followed by lyophilization to obtain the resulting white-colored product. A degree of oxidation (DO) of 380 μmol/g was obtained based on conductometric titration.

#### 2.2.2. Synthesis of cCNCs–PDDA

The coating of PDDA on the surface of cCNCs was achieved non-covalently using a modified reported procedure [39]. Briefly, PDDA (5 mL, 28 wt%, MW 8500) was added to a suspension of cCNCs (2 mL, 5 wt%), and the mixture was sonicated for 30 min, followed by mechanical stirring for 24 h at room temperature. NaCl (0.5 g) was added to the mixture and then stirred for another 24 h at room temperature. The resulting suspension was diluted with deionized water, centrifuged, and then washed with deionized water several times. cCNCs–PDDA were isolated via freeze-drying as a white solid and kept at 4 °C prior to use. The freeze-dried sample was denoted as cCNCs–PDDA-8500. The same procedure was repeated for the preparation of other cCNCs coated with PDDA with MWs of 240,000 or 400,000–500,000 and the samples were denoted as cCNCs–PDDA-240,000 and cCNCs–PDDA-400,000–500,000 respectively.

### 2.3. Characterization of cCNC–PDDA Samples

#### 2.3.1. Fourier Transform Infrared (FTIR) Spectroscopy 

The FTIR spectra of cCNCs and freeze-dried cCNC–PDDA samples were obtained at room temperature using a PerkinElmer FTIR spectrophotometer. About 2% of the CNC samples in well-dried potassium bromide were prepared as pellets. A background measurement was first taken using a neat potassium bromide pellet. A total of 32 scans were recorded for each measurement with spectra in the range of 4000–400 cm^−1^.

#### 2.3.2. Dynamic Light Scattering (DLS) and Zeta Potential

DLS and zeta potential analyses were performed using a Malvern Zetasizer Nano ZS instrument (model: ZEN3600; 4.0 mW helium–neon laser (λ = 633 nm); an avalanche photodiode detector). cCNCs and cCNCs–PDDA 0.05 wt% dispersions in Milli-Q water were used for DLS and the particle concentration was kept at 0.25 wt% for zeta potential measurements. The suspensions were sonicated, filtered through a 0.45 µm filter membrane prior to measurements, and analyses were carried out at 25 °C and neutral pH. The results were measured in triplicate with averages reported.

#### 2.3.3. Elemental Analysis

Elemental analysis data (C, H, N, and S content by mass) for cCNCs and cCNC–PDDA samples were collected on a Thermo Scientific Flash 2000 Organic Elemental Analyzer. The analysis was repeated twice, and the averages were obtained.

#### 2.3.4. Transmission Electron Microscopy (TEM)

TEM images of cCNC–PDDA samples were taken on a Hitachi H-7500 operating at 80 kV in high-resolution mode. cCNC–PDDA samples at a concentration of 0.1 mg/mL were prepared in double distilled water with 15 min bath sonication at room temperature. Then, the sample (6 µL) was loaded on the TEM grid (Cu-300CN, Pacific Grid-Tech, San Francisco, CA, USA) and any excess solution was wiped off using the edge of a wet filter paper. Sample staining was carried out at pH 6.8–7.0 using UranyLess^®^ (Electron Microscopy Sciences (EMS), Hatfield, PA, USA) for 60 s and air-dried in the dark before TEM analysis. All samples were analyzed in the magnification range of 40,000–600,000×.

#### 2.3.5. Thermogravimetric Analysis (TGA) 

TGA of the freeze-dried samples was run on a Netzsch STA 449F1 instrument at a heating rate of 10 °C/min from room temperature to 800 °C under nitrogen purge gas.

### 2.4. Antibacterial Studies

#### 2.4.1. Bacterial Lawn Inhibition Assay

An amount of 100 µL of each 0.1 ABS_600_ culture (SA, EC, and PA) was evenly spread over the surface of sterile 100 mm TSA plates. Then, 10 µL drops of cCNC samples at dilutions of 5, 2.5, 1.25, 0.625, and 0.31 mg/mL were pipetted onto the plate surface in separate sections of a grid (15 drops per plate), with each drop covering a ~6–8 mm diameter circle. After drying, the resulting cCNC concentrations were 1.3, 0.65, 0.32, 0.16, and 0.08 µg/mm^2^ of agar surface. Plates were incubated for 24 h at 35 °C and growth inhibition was observed within each drop area.

#### 2.4.2. Determination of Inhibitory Concentration

The inhibitory concentration values were defined as the lowest concentration of nanoparticles where either no visible bacterial growth was observed (total inhibition) or significant growth inhibition was observed (partial inhibition). Each experiment was repeated five times.

## 3. Results and Discussion

### 3.1. Design, Synthesis, and Characterization of cCNCs–PDDA

**Figure 1:**
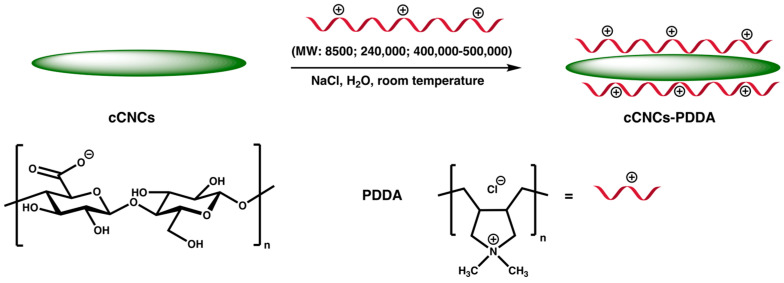
Fabrication of cationic cCNCs–PDDA via non–covalent surface functionalization of cCNCs with PDDA of different molecular weights.

cCNCs used in this study were derived via a one-step oxidation procedure of softwood pulp in the presence of APS at 60 °C for 16 h as previously reported [35,36]. This APS oxidation is a scalable method for forming highly crystalline cCNCs with negative surface charge resulting from the carboxylate groups. The surface of cCNCs was then functionalized with cationic PDDA of varying MWs (8500; 240,000; 400,000–500,000 Da) to obtain the desired cationic cCNC–PDDA samples (Figure 1). The reaction was performed at room temperature in the presence of sodium chloride which helped to promote the ionic interactions between anionic cCNCs and cationic PDDA. The resulting cCNC–PDDA samples were characterized by FTIR spectroscopy, DLS, zeta potentials, elemental analysis, TEM, and TGA.

**Figure 2:**
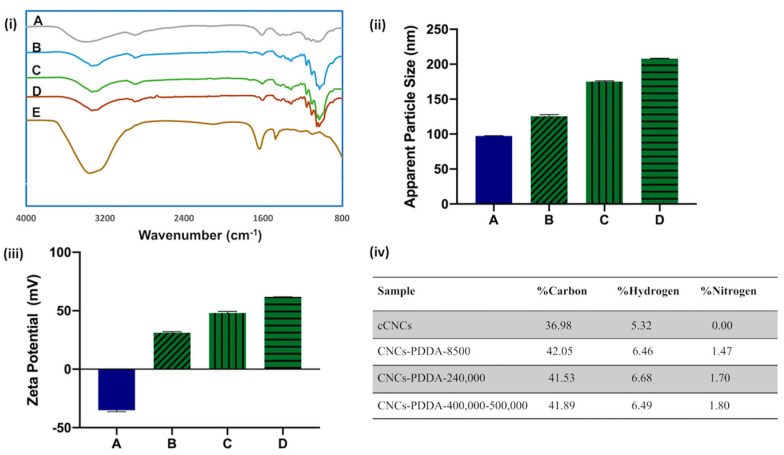
(**i**) FTIR spectra; (**ii**) apparent particle size as measured by DLS; (**iii**) zeta potentials; (**iv**) elemental analysis of cCNCs and cCNC–PDDA samples: (**A**) cCNCs, (**B**) cCNCs–PDDA–8500, (**C**) cCNCs–PDDA-240,000, (**D**) cCNCs–PDDA–400,000–500,000 and (**E**) free PDDA–8500.

FTIR analysis of cCNCs showed typical peaks located around 3200–3600 cm^−1^ (O–H stretching vibration), 2800–3000 cm^−1^ (C–H stretching vibration), 1615 cm^−1^ (C=O stretching vibration of COO-), and 1060–1162 cm^−1^ (C–O and C–O–C stretching vibration) (Figure 2i(A)). For cCNC–PDDA samples, the characteristic stretching bands remained almost the same as for cCNCs, indicating that the coating of PDDA onto cCNCs did not alter the chemical structure of cCNCs (Figure 2i(B–D)). While the FTIR spectrum of free PDDA (Figure 2i(E)) normally displayed a characteristic peak around 1114–1120 cm^−1^ (N–C stretching vibration), this was masked by the cCNC bands in the spectra of cCNC–PDDA samples [39,40].

DLS was used to estimate the hydrodynamic apparent particle size of cCNCs before and after chemical modification with PDDA of varying MWs. Figure 2ii shows that cCNCs had an apparent particle diameter of 97.4 ± 0.4 nm and after surface modification with PDDA, an increase in hydrodynamic apparent particle sizes was observed for all cCNC–PDDA samples (Table S1). The increase in apparent particle size for the cCNC–PDDA samples was a good indication of the presence of PDDA decorated on the surface of cCNCs. The colloidal stability of the cCNC suspensions and the nature of the surface charge on cCNCs and CNC–PDDA samples were assessed by measuring the zeta potentials of the nanoparticles in water at 0.25 wt% concentration (Figure 2iii). cCNCs had a zeta potential of −35.1 ± 1.2 mV due to the presence of the anionic carboxylate (COO-) groups resulting from the APS oxidation of the primary alcohol groups of native cellulose. The negative zeta potential value of cCNCs changed to positive values for all cCNC–PDDA samples which confirmed that the surface of cCNCs was successfully wrapped with cationic PDDA polymers (Figure 2iii; Table S1). The zeta potential values of cCNCs and cCNC–PDDA samples further indicated their highly stable colloidal suspensions as in general, absolute zeta potential values exceeding 30 mV are considered to be highly stable colloidal suspensions [41,42]. Elemental analysis was further used to confirm the successful surface coating of PDDA onto cCNCs with the appearance of nitrogen in all cCNC–PDDA samples (Figure 2iv). We also observed that an increase in MW of the PDDA grafted onto cCNCs led to an increase in hydrodynamic size, zeta potential, and nitrogen content (Figure 2ii–iv).

**Figure 3:**
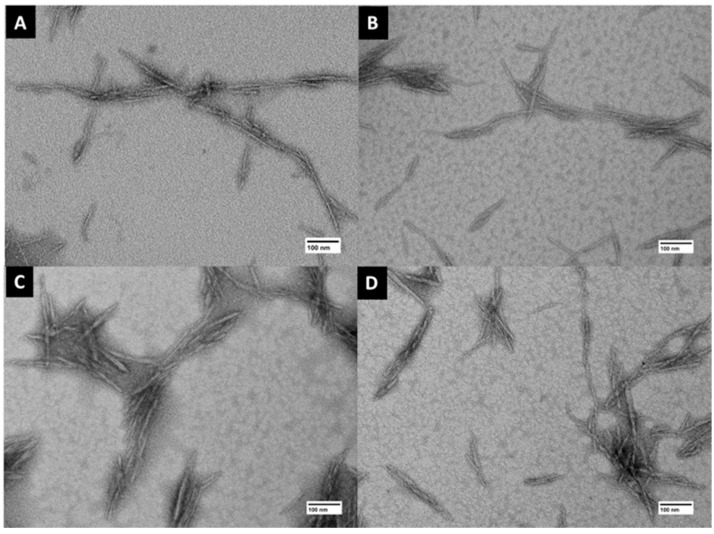
TEM images displaying the morphologies of cCNCs and cCNC–PDDA samples: (**A**) cCNCs, (**B**) cCNCs–PDDA–8500, (**C**) cCNCs–PDDA–240,000, and (**D**) cCNCs–PDDA–400,000–500,000. Scale bars are 100 nm.

The morphologies of the cCNCs and cCNC–PDDA samples were investigated by TEM. TEM images of cCNCs showed rod-shaped geometry and the cCNC-PDDA samples maintained the same morphology even after surface modification with PDDA (Figure 3). cCNCs–PDDA dimensions (length and width) were obtained from the analysis of a minimum of 200 particles with an average length of 133–145 nm and width of 5.09–5.72 nm (Table S2). 

**Figure 4:**
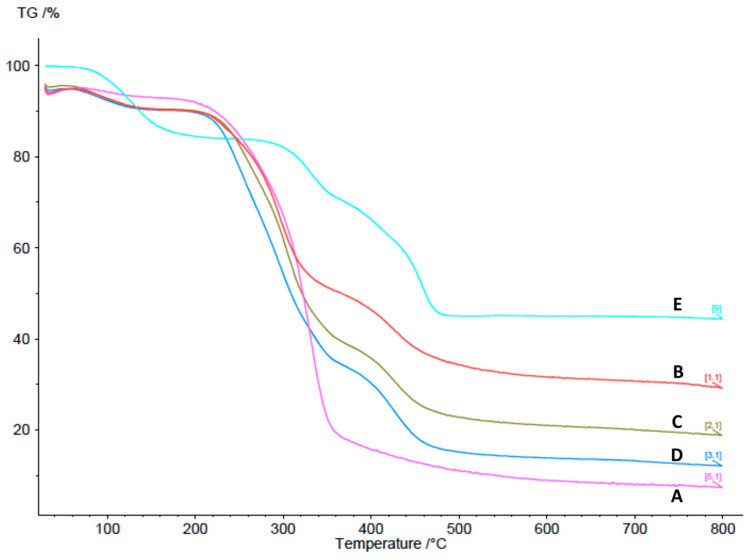
TGA analysis of cCNCs and cCNC–PDDA samples: (**A**) cCNCs, (**B**) cCNCs–PDDA-8500, (**C**) cCNCs–PDDA-240,000, (**D**) cCNCs–PDDA-400,000–500,000, and (**E**) PDDA-400,000–500,000.

The thermal stability of cCNCs, PDDA, and cationic cCNC–PDDA samples was analyzed by TGA (Figure 4). In comparison to cCNCs, the cCNCs–PDDA exhibited greater weight loss in the temperature region of 100 to 140 °C due to loss of water. Further weight loss between 230–460 °C could be attributed to the thermal decomposition of the cCNCs and PDDA [43]. cCNCs were found to have the lowest final residual weight at 800 °C, with increasing weight loss for cCNCs–PDDA from samples B to D.

### 3.2. Assessment of the Antibacterial Properties of Cationic cCNC–PDDA Samples

The antibacterial activities of the synthesized cationic cCNC–PDDA samples were assessed against three model bacterial species, namely *Staphylococcus aureus* (SA, Gram-positive), *Escherichia coli 23934* (EC, Gram-negative), and *Pseudomonas aeruginosa* (PA, Gram-negative). Suspensions of cCNCs–PDDA at 5 mg/mL necessitated brief sonication in distilled water and appeared cloudy. This, combined with the difficulty of recognizing bacterial growth (cloudiness) and the potential of nanoparticles settling out of solution, made traditional minimum inhibitory concentration (MIC) determination challenging. Therefore, the antibacterial activity of the nanomaterials was determined using a bacterial lawn inhibition assay, whereby varying concentrations of cCNC–PDDA conjugates were overlaid on a lawn of bacteria consisting of ~10^7^ cells/plate (10^5^ cells/CNCs drop). As such, these cCNCs–PDDA were no longer in solution, making the term “MIC” imprecise except to indicate initial cCNC concentrations placed on the agar. Effective concentrations were difficult to ascertain (traditional MIC) due to the turbidity of suspended cCNC conjugates, the small sample volume involved, and the difficulty of observing bacterial growth in test wells. In addition, the proposed surface application of cCNCs–PDDA as an antibacterial coating material suggested that a coating/surface assay would be more appropriate for this study. We therefore determined antibacterial activity in a manner that ensured the direct contact of cCNC conjugates with bacterial cells via surface coating. Zones were then observed for complete (no growth, termed MIC^1^) or partial (reduced growth, termed MIC^2^) inhibition. In the surface assay, growth would occur through the formation of colonies/bacterial lawns visible to the naked eye, and concentrations of CNCs insufficient to coat all cells to the point necessary for growth inhibition would only result in partial inhibition. Nonetheless, this value (MIC^2^) is of importance as it recognizes a point of antimicrobial effectiveness often ignored in a broth dilution assay. Although the direct comparison to traditional MIC values was difficult, we provided supplementary data from broth dilution assays we initially conducted (Figure S2).

**Figure 5:**
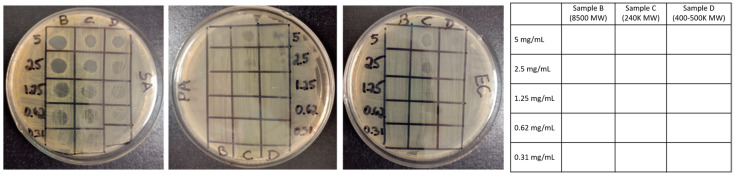
Bacterial lawn growth inhibition assay of (**B**) cCNCs–PDDA-8500, (**C**) cCNCs–PDDA-240,000, and (**D**) cCNCs–PDDA-400,000–500,000 at concentrations of 5.00, 2.50, 1.25, 0.62, and 0.31 mg/mL on TSA plates inoculated with SA, PA, and EC (pictures from left to right, respectively). The table on the right side represents a legend for the petri dish layout. Note: For pristine cCNCs (sample A), refer to Figure S1.

The deposition of cCNC–PDDA conjugates on bacterial lawns (Figure 5) or the inclusion of these in bacterial broth culture (Figure S2) displayed marked antibacterial activity, particularly against Gram-positive bacteria SA. While the Gram-negative pathogens EC and PA were significantly less affected, at least partial growth inhibition was observed in plating assays (Table 1) and broth dilution assays (Figure S2). During this antibacterial study, pristine cCNCs were used as a negative control and it was evident from the bacterial lawn growth inhibition assay that cCNC dispersions at different concentrations (ranging from 0.62 to 5 mg/mL) displayed no antibacterial activity (Figure S1, sample A). However, cationic cCNCs–PDDA (samples B to D) showed antibacterial effects against SA, PA, and EC, with the SA being the most affected in the presence of cCNCs–PDDA of varying MWs (Figure 5). SA was completely inhibited at concentrations of 0.62, 1.25, and 2.50 mg/mL for cationic cCNC–PDDA samples B to D, respectively, and at least partially inhibited by all cCNC–PDDA samples at 0.62 mg/mL (Table 1). Free PDDA was used as a positive control and the results indicated that PDDA possessed antibacterial properties, and that the MW of PDDA had an influence on these antibacterial activities (Table S3). As the MW of PDDA decorated onto cCNCs increased (samples B to D with low, intermediate, and high MW, respectively), antibacterial effectiveness was weakened (Table 1, SA MIC^1^ values increased from samples B to D). This observation was in agreement with previous studies where low MW cationic antimicrobial molecules (synthetic peptide mimics and cationic peptidopolysaccharides) were more effective than high molecular weight molecules [44,45]. However, in the case of Gram-negative bacteria, the MW of PDDA decorated onto cCNCs had a critical effect, with low-MW cCNCs–PDDA (Sample B) being ineffective and only intermediate-MW cCNCs–PDDA (Sample C) showing a moderate effect. PA was the most resistant, with only partial inhibition at 5 mg/mL (sample C) and no inhibition in all other cases. EC exhibited intermediate sensitivity, with sample C being most effective. 

Overall, the Gram-positive bacteria (SA) exhibited stronger antibacterial sensitivity to cationic cCNCs–PDDA when compared to Gram-negative bacteria (PA and EC). This can potentially be attributed to the difference in the cell wall structures of the bacteria and the presence of an outer lipopolysaccharide membrane outside the cytoplasmic (inner) membrane of the Gram-negative bacteria [46]. A recent study reported that CNC/Ag hybrid material could inactivate the cell via an “attacking–attacking” strategy whereby rod-like CNCs could pierce the cell membrane enabling Ag^+^ from the Ag NPs to enter the cell and disrupt vital cellular metabolic processes [47]. Similarly, in our study, we suspected that both the rod-like nature of the CNCs and the cationic nature of the PDDA could contribute to the observed antibacterial effects; however, an in-depth study of the mode of action of the cCNCs–PDDA is required.

## 4. Conclusions

In summary, we reported the fabrication of cationic carboxylated CNCs coated with PDDA of varying molecular weights. The resulting cationic cCNC–PDDA materials were evaluated for their structural, morphological, and thermal properties which confirmed the successful coating of the cationic PDDA polymer onto the anionic cCNCs. A bacterial lawn growth inhibition assay was used to assess the antibacterial effectiveness of the cationic cCNC–PDDA conjugates against SA (Gram-positive), PA, and EC (Gram-negative). cCNCs–PDDA had significant antibacterial activity against SA even at low concentrations (0.62 mg/mL). cCNCs coated with the low molecular weight PDDA (sample B) were most effective against SA, while only the cCNCs decorated with intermediate molecular weight PDDA displayed some effectiveness against the Gram-negative bacteria. Overall, cationic cCNCs–PDDA could be promising candidates for sustainably-produced, antibacterial materials as the APS process can be used to upcycle a variety of different lignocellulosic biomass residues in cCNCs, with potential end-user applications such as antibacterial surfaces or coatings for the resulting cCNC–PDDA conjugates. Future biological studies regarding the mode of action of the cCNCs–PDDA will be required, in particular for understanding the specificity of cCNCs–PDDA’s bactericidal properties towards Gram-positive bacteria.

## Figures and Tables

**Table 1:** MIC values of cCNC–PDDA samples from bacterial lawn inhibition assays (*n* = 5): (**A)** cCNCs, (**B**) cCNCs–PDDA-8500, (**C**) cCNCs–PDDA-240,000 and (**D**) cCNCs–PDDA-400,000–500,000 against the bacterial species (SA, EC, and PA).

	MIC^1^ (mg/mL) (Complete Inhibition)			MIC^2^ (mg/mL) (Partial Inhibition)		
	SA	EC	PA	SA	EC	PA
A	-	-	-	-	-	-
B	0.62	-	-	0.62	-	-
C	1.25	3.50 *	-	0.62	2.50	5.00
D	2.50	5.00	-	0.62	2.50	-

* Average of five MIC^1^ values.

## Data Availability

Data available on request.

## Supplementary Materials

The following supporting information can be downloaded at: https://www.mdpi.com/article/10.3390/polym15040865/s1, Table S1: apparent particle size, polydispersity index, and zeta potentials of cCNCs and cCNCs–PDDA; Table S2: dimensions (average length and width) of cCNC–PDDA samples from TEM images; Table S3: MIC values of free PDDA as positive control; Figure S1: bacterial lawn growth inhibition assay of pristine cCNCs; Figure S2: broth dilution assay of cCNCs and cCNCs–PDDA.
