# Supplementary material for: Carboxylated Cellulose Nanocrystals Decorated with Varying Molecular Weights of Poly(diallyldimethylammonium chloride) as Sustainable Antibacterial Agents
Source: Polymers (Basel). 2023 Feb 9;15(4):865. doi: 10.3390/polym15040865 (PMC9966959; doi:10.3390/polym15040865)
Supplement: Supplementary file 1 [file polymers-15-00865-s001.zip › polymers-2115055-supplementary.pdf]

polymers

MDPI

## Supporting Information

# Carboxylated Cellulose Nanocrystals Decorated with Varying Molecular Weights of Poly(diallyldimethylammonium chloride) as Sustainable Antibacterial Agents

**Eliskander Rabia [1], Beza Tuga [1], José de Ondarza [2], Saleen M. Ramos [1], Edmond Lam [3,4], Sabahudin Hrapovic [3], Yali Liu [3] and Rajesh Sunasee [1,*]**

[1] Department of Chemistry and Biochemistry, State University of New York at Plattsburgh, Plattsburgh, NY 12901, USA; erabi003@plattsburgh.edu (E.R.); btuga001@plattsburgh.edu (B.T.); sramo009@plattsburgh.edu (S.M.R.)
[2] Department of Biological Sciences, State University of New York at Plattsburgh, Plattsburgh, NY 12901, USA; deondaj@plattsburgh.edu
[3] Aquatic and Crop Resource Development Research Centre, National Research Council Canada, Montreal, QC H4P 2R2, Canada; edmond.lam@cnrc-nrc.gc.ca (E.L.); sabahudin.hrapovic@cnrc-nrc.gc.ca (S.H.); yali.liu@cnrc-nrc.gc.ca (Y.L.)
[4] Department of Chemistry, McGill University, 801 Sherbrooke St. West, Montreal, QC H3A 0B8, Canada
* Correspondence: rajesh.sunasee@plattsburgh.edu; Tel.: +1-518-564-2703

**Table S1.** Apparent particle size (DLS), polydispersity index and zeta potential of cCNCs and cCNCs-PDDA samples.

| Sample | Apparent Particle Size (nm) | Polydispersity Index (PDI) | Zeta Potential (mV) |
|---|---|---|---|
| cCNCs | 97.4 ± 0.4 | 0.21 | -35.1 ± 1.2 |
| cCNCs-PDDA-8500 | 125.6 ± 2.2 | 0.18 | +31.2 ± 0.8 |
| cCNCs-PDDA-240,000 | 175.2 ± 0.9 | 0.27 | +48.0 ± 1.4 |
| cCNCs-PDDA-400,000–500,000 | 207.9 ± 0.6 | 0.26 | +61.8 ± 0.1 |

**Table S2.** Dimensions (average length and width) of cCNCs-PDDA samples from TEM images.

| Sample | Length (nm) | Width (nm) | # of Particles |
|---|---|---|---|
| cCNCs-PDDA-8500 | 133.1 ± 37.5 | 5.26 ± 1.03 | 218 |
| cCNCs-PDDA-240,000 | 145.3 ± 39.1 | 5.09 ± 1.23 | 240 |
| cCNCs-PDDA-400,000–500,000 | 133.7 ± 37.1 | 5.72 ± 1.79 | 240 |

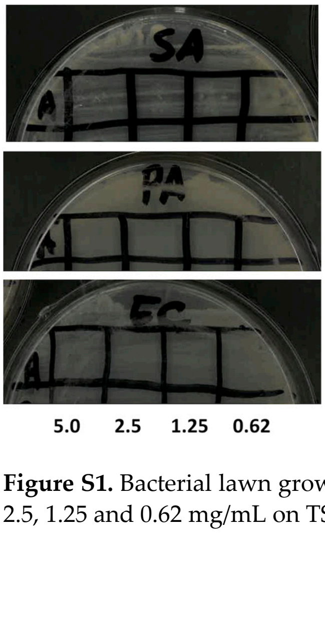


**Figure S1.** Bacterial lawn growth inhibition assay of pristine cCNCs (Sample A) at dilutions of 5.0, 2.5, 1.25 and 0.62 mg/mL on TSA plates inoculated with SA, EC and PA.

## S2. Antibacterial test

*MIC method:* 24 h broth cultures of SA, EC and PA in Tryptic Soy Broth (TSB; Hardy Diagnostics, Santa Maria, CA) were grown at 30 ˚C and diluted to an absorbance of 0.1 (600 nm) in TSB, except that a 0.2 $ABS_{600}$ culture was used for the initial cCNCs dilution step (2.5 mg/mL). 100 µL aliquot of each culture was pipetted into wells of sterile 96 well assay plates. 100 µL of each cCNCs stock solution (5 mg/mL) was added to the first well containing bacterial culture (0.2 $ABS_{600}$) and mixed by repeated pipetting (final concentration = 2.5 mg/mL cCNCs and 0.1 A600 of cells); 100 µL of this mixture was then transferred to the next well (0.1 $A_{600}$ culture) and mixed, and this process repeated to obtain concentrations of 2.50, 1.25, 0.63, 0.31, 0.16 and 0.08 mg/mL of the cCNC materials. Wells were incubated for 24 h at 35° C and evaluated for growth (turbidity) and survival (streaking 5 µL of each well onto agar plates).

*Results:* Serial dilution of different cCNCs solutions attained concentrations of 2.5–0.08 mg/mL in culture containing ~$10^7$ cells/mL. Dilutions of 2.5 and 1.25 mg/mL exhibited turbidity due to cCNCs. Following incubation at 30 °C for 24 h, cloudiness of each well due to bacterial growth was assessed. Absence of cloudiness indicated growth inhibition.

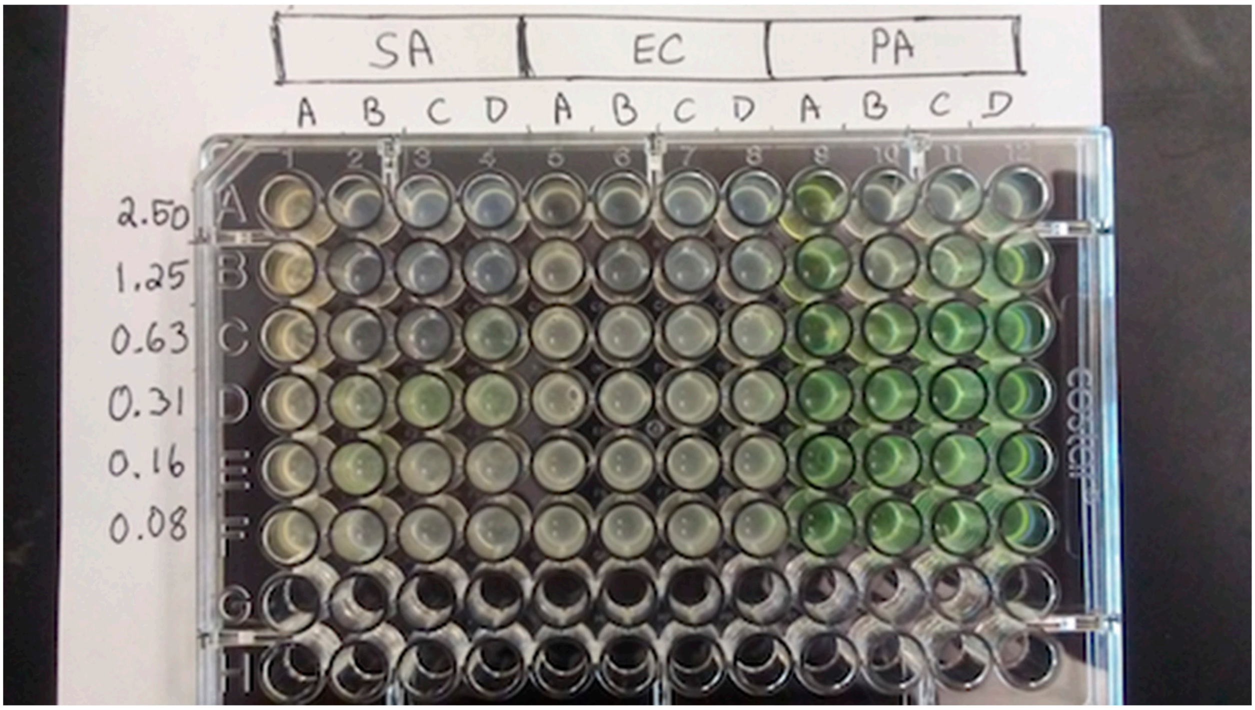


**Figure S2.** Broth dilution assay with (A) cCNCs, (B) cCNCs-PDDA-8500, (C) cCNCs-PDDA-240,000, and (D) cCNCs-PDDA-400,000–500,000 against the bacterial species (SA, EC, PA) at varying concentrations.

**Table S3.** MIC values of free PDDA as positive control from bacterial lawn inhibition assays.

| Bacteria | PDDA-8500 | PDDA-400–500k |
|---|---|---|
| *E. coli* | 0.625 mg/mL | 0.078 mg/mL |
| *S. aureus* | 1.25 mg/mL | 0.156 mg/mL |
| *P. aeruginosa* | none | 2.5 mg/mL |

**Disclaimer/Publisher's Note:** The statements, opinions and data contained in all publications are solely those of the individual author(s) and contributor(s) and not of MDPI and/or the editor(s). MDPI and/or the editor(s) disclaim responsibility for any injury to people or property resulting from any ideas, methods, instructions or products referred to in the content.
